# Combination of memory type ratio and product estimators under extended EWMA statistic with application to wheat production

**DOI:** 10.1038/s41598-023-40687-4

**Published:** 2023-08-20

**Authors:** Rashiqa Zahid, Muhammad Noor-ul-Amin, Imad Khan, Salman A. AlQahtani, Pranav Kumar Pathak, Javed Rahimi

**Affiliations:** 1 COMSATS University Islamabad-Lahore Campus, Lahore, Pakistan; 2 Department of Statistics, Abdul Wali Khan University Mardan, Mardan, Pakistan; 3 Department of Computer Engineering, College of Computer and Information Sciences, King Saud University, P.O. Box 51178, 11543 Riyadh, Saudi Arabia; 4 School of Continuing Studies, McGill University, Quebec, Canada; 5 Kabul City Agriculture and Food Processing Institute, Kabul, Afghanistan

**Keywords:** Energy science and technology, Materials science, Physics

## Abstract

The extended exponential weighted moving average (EEWMA) statistic is a memory type statistic that uses past observations along with the current information for the estimation of a population parameter to improve the efficiency of the estimators. This study utilized the EEWMA statistic to estimate the population mean with a suitable auxiliary variable. The ratio and product estimators are proposed for the surveys that are time-based by using current information along with that information. The approximate mean square errors are computed for the proposed memory type estimators and mathematical comparison is discussed to demonstrate the efficiency of the estimator. The simulation study was carried out to evaluate the performance of the proposed memory type estimators. It can be seen from the results that the efficiency of the estimator enhances by utilizing the current sample as well as past information. A real-life example is presented to illustrate the usage of proposed estimators.

## Introduction

The efficiency of ratio or product type estimators can be improved by using auxiliary information. Auxiliary information has been considered in education, biostatistics, medical research, agriculture, etc. For example, the tree diameter can be used as an auxiliary variable in an agricultural survey to estimate the average amount of timber produced by the tree. When the linear relationship between the study and auxiliary variable is highly positive with the line passing through the origin, then ratio estimator is used. The ratio estimator is defined by Cochran (1940)^1^ as

1 $${\widehat{\overline{Y}} }_{r}= \frac{\overline{y} }{\overline{x}}{\mu }_{x},$$

where it is assumed that the population mean ${\mu }_{x}$ of the auxiliary variable is known in advance. Here $\overline{y }$ is the sample mean of the study variable, $\overline{x }$ is the sample mean of the auxiliary variable. When there is a negative linear relationship between the study and auxiliary variable, it is convenient to use the product estimators. The product estimator by Robson (1957)^2^ is given by

2 $${\widehat{\overline{Y}} }_{p}= \frac{\overline{y}}{{\mu }_{x}}\overline{x }.$$

The approximate mean square errors (MSEs) are

3 $$MSE\left({\widehat{\overline{Y}} }_{r}\right)\approx \theta \left[{C}_{y}^{2}+ {C}_{x}^{2}-2\rho {C}_{y}{C}_{x}\right],$$

and

4 $$MSE\left({\widehat{\overline{Y}} }_{p}\right)\approx \theta \left[{C}_{y}^{2}+ {C}_{x}^{2}+2\rho {C}_{y}{C}_{x}\right],$$

where θ = (*1–n⁄N*)*/n*, *n* is the sample size, *N* is the population size, ${C}_{y}$ and ${C}_{x}$ are the coefficient of variation for study and auxiliary variable, respectively. The coefficient of variation is a statistical measure used to assess the relative variability between the study variable and the auxiliary variable. Additionally, the correlation coefficient (*ρ*) quantifies the strength and direction of the linear relationship between the study and auxiliary variables. In the field of research, several authors, including Grover, Kaur, and Vishawkarma^3^, Noor-Ul-Amin, Shahbaz, and Kadilar^4^, Zaman^5^, Zaman and Bulut^6^, Zaman^7^, Yasmeen, Noor-Ul-Amin, and Hanif^8^, Yadav and Zaman^9^, Zaman and Kadilar^10^ and Irfan et al.^11^, have explored the utilization of auxiliary information to develop estimators for population parameters. These researchers have recognized the potential benefits of incorporating auxiliary information in the estimation process, aiming to enhance the efficiency and accuracy of population parameter estimation.

The memory type ratio and product estimators were initially proposed by Noor-Ul-Amin^12^ and Noor-Ul-Amin^13^ as an improvement over existing estimators. The author introduced the memory type ratio estimator utilizing the Exponentially Weighted Moving Average (EWMA) statistic, while Noor-Ul-Amin^13^ introduced the memory type product estimator using the Hybrid Exponentially Weighted Moving Average (HEWMA) statistic. The EWMA statistic was originally introduced by Roberts (1959) ^14^ as a tool to monitor changes in the process mean. It is a statistic that incorporates both the current and past samples to observe the change in the mean over time. The EWMA statistic can be defined as follows:

5 $${Z}_{i}=\lambda \overline{y }+\left(1-\lambda \right){Z}_{i-1},$$

where $\overline{y }$ is the current sample mean of observation in time $i=1, 2, \dots$ The smoothing parameter (λ) is a value between 0 and 1 that determines the weighting given to current and past observations in the EWMA statistic. A larger value of λ assigns more weight to the current values and less weight to past observations. Conversely, a smaller value of λ gives more weight to past observations and less weight to the current data.When λ is equal to 1, the EWMA statistic becomes equivalent to the usual sample mean, and the latest observation receives all the weight. In this case, there is no consideration given to past observations, and the estimate is solely based on the most recent data point. The term ${Z}_{i-1}$ is used to represent past observations. The expected mean of the prior sample is taken as its initial value i.e. ${Z}_{0}$. When the initial value is not available, it can be obtained from the pilot survey.

We have,

6 $$E\left({Z}_{t}\right)= {\mu }_{y},$$

where ${\mu }_{y}$ is the mean and ${\sigma }_{y}^{2}$ is the variance of the study variable. The limiting form of variance of EWMA statistic is given by

7 $$Var\left({Z}_{t}\right)= \frac{{\sigma }_{y}^{2}}{n}\left[\frac{\lambda }{2-\lambda }\right].$$

The respective memory type ratio and product estimators due to Noor-Ul-Amin^13^) are

8 $${\widehat{t}}_{rmi}= \frac{{Z}_{t}}{{Q}_{t}}{\mu }_{x},$$

and

9 $${\widehat{t}}_{pmi}= \frac{{Z}_{t}}{{\mu }_{x}}{Q}_{t},$$

where ${Z}_{t}$ and ${Q}_{t}$ are the memory type statistic for both variables.The respective MSEs are given by

$$MSE\left({\widehat{t}}_{rmi}\right)\approx \theta \frac{\lambda }{2-\lambda }\left[{C}_{y}^{2}+{C}_{x}^{2}-2\rho {C}_{y}{C}_{x}\right],$$

and

$$MSE\left({\widehat{t}}_{pmi}\right)\approx \theta \frac{\lambda }{2-\lambda }\left[{C}_{y}^{2}+{C}_{x}^{2}+2\rho {C}_{y}{C}_{x}\right].$$

The proposed estimators hold particular relevance for surveys conducted at regular time intervals, such as the Pakistan Social and Living Standard Measurement (PSLM) survey, which is a routine activity conducted by the Pakistan Bureau of Statistics. Additionally, the Pakistan government conducts the Labor Force Survey annually through the platform provided by the Pakistan Bureau of Statistics. These surveys, including economic and health surveys, provide essential insights into socio-economic factors over time. By incorporating the extended EWMA statistic and leveraging both past and current information, the proposed estimators offer a robust framework for estimating population parameters with enhanced precision. This framework is expected to outperform previous estimators used in time-scaled surveys. Consequently, researchers, policymakers, and stakeholders can make more informed decisions based on reliable and up-to-date information. The proposed methodology has the potential to significantly improve the performance of estimators in various time-scaled surveys, contributing to the accuracy and usefulness of survey results.

Let $T_{1} ,T_{2} ,.....,T_{i} ,..$ be a sequence of random variables with mean $\mu$ and variance $\sigma^{2}$ taken from a normal population, then the EEWMA statistic with smoothing constants ${\lambda }_{1}$ and ${\lambda }_{2}$ given by Naveed et al.^15^ is defined as

10 $${Z}_{i0}= {\lambda }_{1}{T}_{i}- {\lambda }_{2}{T}_{i-1}+(1-{\lambda }_{1}+{\lambda }_{2}){Z}_{i0-1,}$$

The expected mean of EEWMA statistic is given by

$$E\left({Z}_{i0}\right)=\mu ,$$

and the variance is

11 $$Var\left({Z}_{i0}\right)= {\sigma }^{2}\gamma ,$$

where $$\gamma =\left\{\frac{\left({\lambda }_{1}^{2}+ {\lambda }_{2}^{2}\right)-2a{\lambda }_{1}{\lambda }_{2}}{2\left({\lambda }_{1}-{\lambda }_{2}\right)-{\left({\lambda }_{1}-{\lambda }_{2}\right)}^{2}}\right\}$$

This research paper encompasses various sections that delve deeper into the proposed estimators and their performance. In Sect. “Proposed memory type estimators”, the construction of the proposed estimators is discussed. Section “Simulation study” is based on the simulation study and the main findings from the simulation results are presented in Sect. “Discussion”. Section “Mathematical comparison” is the mathematical comparison of the proposed estimators with the existing estimators and the real data application is given in Sect. “Real data application”. Finally, the conclusion is presented in Sect. “Conclusion”.

## Proposed memory type estimators

The literature is rich with the conventional ratio and product estimators that only use the current sample information. As we know, surveys are frequently carried out at regular intervals. This allows for the utilization of both historical sample data and present information. Our goal is to bridge the gap using estimators that incorporate not only the current sample information but also the data from past samples. In this context, we introduce the memory type ratio and product estimators that incorporate both the current and past sample information. By utilizing additional historical data, these estimators offer improved performance over the conventional ones. For the said objective, we used the Extended Exponentially Weighted Moving Average (EEWMA) statistic introduced by Naveed et al.^15^. In our methodology, we consider a variable of interest denoted as *y* and an auxiliary variable denoted as *x*. At each time point *t*, we calculate the EEWMA statistic for both *y* and *x*. This EEWMA statistic serves as a crucial component in formulating the memory type ratio estimators.

12 $${Z}_{ie}= {\lambda }_{1}\overline{y }- {\lambda }_{2}{\overline{y} }_{t-1}+(1-{\lambda }_{1}+{\lambda }_{2}){Z}_{ie-1,}$$

and

13 $${Q}_{ie}= {\lambda }_{1}\overline{x }- {\lambda }_{2}{\overline{x} }_{t-1}+(1-{\lambda }_{1}+{\lambda }_{2}){Q}_{ie-1,}$$

where $0<{\lambda }_{1}<1$ and $0<{\lambda }_{2}<{\lambda }_{1}$, and ${\lambda }_{1}=\lambda$ for EWMA statistic.

The initial values can be set as zero, which is a common practice. However, in some cases, it may be beneficial to estimate the expected mean from a pilot survey or any preliminary data available. The proposed estimators are given by the following formulas

14 $${\widehat{t}}_{ermi}= \frac{{Z}_{ie}}{{Q}_{ie}}{\mu }_{x},$$

and

15 $${\widehat{t}}_{epmi}= \frac{{Z}_{ie}}{{\mu }_{x}}{Q}_{ie},$$

respectively, where ${\mu }_{x}$ is the population mean of the auxiliary variable, it is assumed to be known in advance. The mean square expression for the proposed memory type estimator using Taylor series approximation is obtained by the following notations,

if ${e}_{y}= \frac{{Z}_{ie}-{\mu }_{y}}{{\mu }_{y}}$ and ${e}_{x}= \frac{{Q}_{ie}-{\mu }_{x}}{{\mu }_{x}},$ then we have

$${E(e}_{y})={E(e}_{x})=0 \; and \; \theta =\frac{1}{n}-\frac{1}{N},$$

$$E\left({e}_{y}^{2}\right)=\theta \frac{Var({Z}_{ie})}{{{\mu }_{y}}^{2}}= \theta \frac{{\sigma }_{y}^{2}}{{{\mu }_{y}}^{2}}\left[\gamma \right]$$

16 $$E\left({e}_{x}^{2}\right)=\theta \frac{Var({Q}_{ie})}{{{\mu }_{x}}^{2}}= \theta \frac{{\sigma }_{x}^{2}}{{{\mu }_{x}}^{2}}\left[\gamma \right],$$

$$E\left({e}_{x}{e}_{y}\right)=\frac{Cov({Z}_{ie},{Q}_{ie})}{{\mu }_{y}{\mu }_{x}}= \theta \rho {C}_{y}{C}_{x}\left[\gamma \right].$$

The MSE expression for the proposed memory type ratio estimator is simplified as

$${\widehat{t}}_{ermi}= {\mu }_{y}\left(1+{e}_{y}\right){\left(1+{e}_{x}\right)}^{-1}.$$

$$MSE\left({\widehat{t}}_{ermi}\right)\approx {\mu }_{y}\left(1+{e}_{y}\right)\left(1-{e}_{x}\right),$$

$$MSE\left({\widehat{t}}_{ermi}\right)\approx \theta \left[\frac{Var\left({Z}_{ie}\right)}{{{\mu }_{y}}^{2}}+\frac{Var\left({Q}_{ie}\right)}{{{\mu }_{x}}^{2}}-2\frac{Cov\left({Z}_{ie},{Q}_{ie}\right)}{{\mu }_{y}{\mu }_{x}}\right],$$

or

17 $$MSE\left(\widehat{t}{}_{ermi}\right)\approx \theta \gamma \left[{C}_{y}^{2}+{C}_{x}^{2}-2\rho {C}_{y}{C}_{x}\right].$$

or

18 $$MSE\left({\widehat{t}}_{epmi}\right)\approx \theta \gamma \left[{C}_{y}^{2}+{C}_{x}^{2}+2\rho {C}_{y}{C}_{x}\right].$$

## Simulation study

To assess the effectiveness of the proposed ratio and product estimators, a simulation study was conducted. The performance of the proposed estimator, denoted as $\left({\widehat{t}}_{ermi}\right)$, was compared to the previous ratio estimator $\left({\widehat{t}}_{rmi}\right)$ developed by Noor-Ul-Amin^13^. The evaluation involved computing mean square errors and relative efficiencies based on 50,000 replications. The mean square error was determined using the following formula:

19 $$MSE\left({t}_{i}\right)= \frac{1}{50000}\sum_{i=1}^{50000}{({t}_{i}-{\mu }_{y})}^{2},$$

where $$t_{i} = \overline{y},\overset{\lower0.5em\hbox{$\smash{\scriptscriptstyle\frown}$}}{t}_{ermi} ,\overset{\lower0.5em\hbox{$\smash{\scriptscriptstyle\frown}$}}{t}_{epmi} ,\overset{\lower0.5em\hbox{$\smash{\scriptscriptstyle\frown}$}}{t}_{rmi} ,\overset{\lower0.5em\hbox{$\smash{\scriptscriptstyle\frown}$}}{t}_{pmi} .$$ The relative efficiency is calculated by using the formula:

20 $$RE\left({t}_{i}\right)= \frac{MSE(\overline{y })}{MSE({t}_{i})},$$

The values of MSE for ratio estimators are given in Table 1. The values regarding the REs are presented in Table 2. The values of MSE and RE are computed for several values of a correlation coefficient. i.e. 0.05, 0.25, 0.50, 0.75, and 0.95. The different values of i.e. 0.05, 0.25, 0.50, 0.75, and 1.0 have been used to check the impact of the smoothing constant by fixing ${\lambda }_{2}$.

**Table 1:** MSEs of ratio estimators for ${\lambda }_{2}$=0.03.

$\lambda_{1}$		$0.1$		$0.15$		$0.25$		$0.5$		$0.75$		$1$	
$$\rho$$	$$n$$	$$\overset{\lower0.5em\hbox{$\smash{\scriptscriptstyle\frown}$}}{t}_{rmi}$$	$$\overset{\lower0.5em\hbox{$\smash{\scriptscriptstyle\frown}$}}{t}_{ermi}$$	$$\overset{\lower0.5em\hbox{$\smash{\scriptscriptstyle\frown}$}}{t}_{rmi}$$	$$\overset{\lower0.5em\hbox{$\smash{\scriptscriptstyle\frown}$}}{t}_{ermi}$$	$$\overset{\lower0.5em\hbox{$\smash{\scriptscriptstyle\frown}$}}{t}_{rmi}$$	$$\overset{\lower0.5em\hbox{$\smash{\scriptscriptstyle\frown}$}}{t}_{ermi}$$	$$\overset{\lower0.5em\hbox{$\smash{\scriptscriptstyle\frown}$}}{t}_{rmi}$$	$$\overset{\lower0.5em\hbox{$\smash{\scriptscriptstyle\frown}$}}{t}_{ermi}$$	$$\overset{\lower0.5em\hbox{$\smash{\scriptscriptstyle\frown}$}}{t}_{rmi}$$	$$\overset{\lower0.5em\hbox{$\smash{\scriptscriptstyle\frown}$}}{t}_{ermi}$$	$$\overset{\lower0.5em\hbox{$\smash{\scriptscriptstyle\frown}$}}{t}_{rmi}$$	$$\overset{\lower0.5em\hbox{$\smash{\scriptscriptstyle\frown}$}}{t}_{ermi}$$
0.05	10	0.0054	0.0040	0.0084	0.0071	0.0142	0.0131	0.0341	0.0332	0.0609	0.0606	0.1023	0.1023
	20	0.0026	0.0023	0.0041	0.0035	0.0072	0.0067	0.0171	0.0168	0.0308	0.0307	0.0511	0.0511
	30	0.0018	0.0013	0.0027	0.0023	0.0050	0.0045	0.0112	0.0110	0.0204	0.0204	0.0344	0.0344
	50	0.0010	0.0008	0.0016	0.0014	0.0030	0.0026	0.0068	0.0067	0.0121	0.0121	0.0203	0.0203
	200	0.0002	0.0002	0.0004	0.0003	0.0007	0.0006	0.0017	0.0016	0.0030	0.0030	0.0050	0.0050
	500	0.0001	0.0000	0.0001	0.0001	0.0003	0.0002	0.0006	0.0006	0.0012	0.0012	0.0020	0.0020
0.25	10	0.0050	0.0038	0.0076	0.0065	0.0133	0.0123	0.0314	0.0307	0.0563	0.0561	0.0951	0.0951
	20	0.0024	0.0018	0.0039	0.0033	0.0067	0.0062	0.0159	0.0155	0.0282	0.0281	0.0471	0.0471
	30	0.0016	0.0012	0.0025	0.0021	0.0044	0.0041	0.0105	0.0102	0.0189	0.0188	0.0314	0.0314
	50	0.0009	0.0007	0.0015	0.0012	0.0027	0.0024	0.0062	0.0061	0.0112	0.0112	0.0186	0.0186
	200	0.0002	0.0001	0.0004	0.0003	0.0006	0.0006	0.0015	0.0015	0.0028	0.0028	0.0046	0.0046
	500	0.0000	0.0000	0.0001	0.0001	0.0002	0.0002	0.0006	0.0006	0.0011	0.0011	0.0018	0.0018
0.50	10	0.0043	0.0032	0.0067	0.0056	0.0118	0.0109	0.0281	0.0276	0.0503	0.0501	0.0833	0.0833
	20	0.0021	0.0015	0.0034	0.0029	0.0060	0.0055	0.0147	0.0137	0.0252	0.0251	0.0418	0.0418
	30	0.0015	0.0011	0.0023	0.0019	0.0040	0.0037	0.0092	0.0090	0.0167	0.0166	0.0279	0.0279
	50	0.0008	0.0006	0.0013	0.0011	0.0024	0.0022	0.0056	0.0054	0.0100	0.0099	0.0168	0.0168
	200	0.0002	0.0001	0.0003	0.0002	0.0006	0.0005	0.0014	0.0013	0.0025	0.0025	0.0041	0.0041
	500	0.0000	0.0000	0.0001	0.0001	0.0002	0.0002	0.0005	0.0005	0.0009	0.0009	0.0016	0.0016
0.75	10	0.0040	0.0030	0.0058	0.0049	0.0104	0.0096	0.0247	0.0242	0.0443	0.0441	0.0750	0.0750
	20	0.0020	0.0015	0.0030	0.0026	0.0053	0.0049	0.0124	0.0121	0.0220	0.0220	0.0370	0.0370
	30	0.0013	0.0009	0.0020	0.0017	0.0035	0.0032	0.0081	0.0079	0.0148	0.0147	0.0245	0.0245
	50	0.0007	0.0005	0.0011	0.0010	0.0020	0.0019	0.0050	0.0048	0.0088	0.0087	0.0146	0.0146
	200	0.0002	0.0001	0.0003	0.0002	0.0005	0.0004	0.0012	0.0012	0.0022	0.0022	0.0036	0.0036
	500	0.0000	0.0000	0.0001	0.0001	0.0002	0.0001	0.0005	0.0004	0.0008	0.0008	0.0014	0.0014
0.95	10	0.0034	0.0025	0.0053	0.0045	0.0094	0.0087	0.0219	0.0215	0.0399	0.0397	0.0662	0.0662
	20	0.0031	0.0012	0.0027	0.0023	0.0047	0.0043	0.0109	0.0107	0.0197	0.0197	0.0327	0.0327
	30	0.0011	0.0008	0.0018	0.0015	0.0031	0.0029	0.0073	0.0071	0.0131	0.0130	0.0219	0.0219
	50	0.0007	0.0005	0.0010	0.0009	0.0018	0.0017	0.0043	0.0042	0.0079	0.0079	0.0131	0.0131
	200	0.0001	0.0001	0.0002	0.0002	0.0004	0.0004	0.0010	0.0010	0.0019	0.0019	0.0032	0.0032
	500	0.0000	0.0000	0.0001	0.0000	0.0001	0.0001	0.0004	0.0004	0.0008	0.0007	0.0013	0.0013

**Table 2:** Relative efficiencies of ratio estimators for ${\lambda }_{2}$=0.03.

$\lambda_{1}$		$0.1$		$0.15$		$0.25$		$0.5$		$0.75$		$1$	
**Ρ**	**n**	$$\overset{\lower0.5em\hbox{$\smash{\scriptscriptstyle\frown}$}}{t}_{rmi}$$	$$\overset{\lower0.5em\hbox{$\smash{\scriptscriptstyle\frown}$}}{t}_{ermi}$$	$$\overset{\lower0.5em\hbox{$\smash{\scriptscriptstyle\frown}$}}{t}_{rmi}$$	$$\overset{\lower0.5em\hbox{$\smash{\scriptscriptstyle\frown}$}}{t}_{ermi}$$	$$\overset{\lower0.5em\hbox{$\smash{\scriptscriptstyle\frown}$}}{t}_{rmi}$$	$$\overset{\lower0.5em\hbox{$\smash{\scriptscriptstyle\frown}$}}{t}_{ermi}$$	$$\overset{\lower0.5em\hbox{$\smash{\scriptscriptstyle\frown}$}}{t}_{rmi}$$	$$\overset{\lower0.5em\hbox{$\smash{\scriptscriptstyle\frown}$}}{t}_{ermi}$$	$$\overset{\lower0.5em\hbox{$\smash{\scriptscriptstyle\frown}$}}{t}_{rmi}$$	$$\overset{\lower0.5em\hbox{$\smash{\scriptscriptstyle\frown}$}}{t}_{ermi}$$	$$\overset{\lower0.5em\hbox{$\smash{\scriptscriptstyle\frown}$}}{t}_{rmi}$$	$$\overset{\lower0.5em\hbox{$\smash{\scriptscriptstyle\frown}$}}{t}_{ermi}$$
0.05	10	18.44	24.84	11.87	14.04	6.95	7.53	2.92	2.98	1.63	1.64	0.98	0.98
	20	18.81	25.21	12.05	14.23	6.94	7.51	2.93	2.98	1.63	1.63	0.98	0.98
	30	18.06	24.08	12.17	14.33	6.76	7.30	2.95	3.01	1.64	1.65	0.98	0.98
	50	18.78	25.21	11.91	14.06	6.87	7.44	2.92	2.98	1.63	1.64	0.98	0.98
	200	17.90	23.86	12.20	14.44	6.89	7.46	2.96	3.02	1.64	1.64	0.98	0.98
	500	18.69	25.15	11.78	13.89	6.82	7.38	2.94	3.00	1.63	1.64	0.98	0.98
0.25	10	19.88	26.52	13.04	15.37	7.42	8.02	3.19	3.26	1.77	1.77	1.06	1.06
	20	20.75	27.63	12.93	15.22	7.43	8.04	3.17	3.24	1.77	1.78	1.07	1.07
	30	20.42	27.28	12.78	15.10	7.35	7.94	3.19	3.25	1.77	1.78	1.06	1.06
	50	19.92	26.63	13.14	15.50	7.42	8.02	3.20	3.26	1.76	1.77	1.06	1.06
	200	20.91	28.10	13.34	15.79	7.53	8.15	3.19	3.26	1.77	1.78	1.06	1.06
	500	20.78	27.95	13.40	15.84	7.51	8.14	3.19	3.25	1.78	1.79	1.06	1.06
0.50	10	23.00	30.66	14.91	17.67	8.38	9.08	3.55	3.62	1.98	1.99	1.18	1.18
	20	23.01	30.97	14.46	17.08	8.26	8.94	3.59	3.66	1.99	1.99	1.19	1.19
	30	22.17	29.52	14.57	17.22	8.32	9.01	3.58	3.66	1.98	1.99	1.19	1.19
	50	22.67	30.27	14.58	17.21	8.37	9.06	3.58	3.65	1.99	2.00	1.19	1.19
	200	22.60	30.03	14.94	17.66	8.40	9.08	3.56	3.63	1.99	2.00	1.19	1.19
	500	22.83	30.65	15.00	17.76	8.34	9.02	3.59	3.66	1.98	1.99	1.19	1.19
0.75	10	24.81	33.09	16.93	20.09	9.60	10.40	4.06	4.15	2.24	2.25	1.34	1.34
	20	24.86	33.04	16.39	19.32	9.41	10.19	4.05	4.13	2.25	2.26	1.35	1.35
	30	25.41	33.82	16.37	19.36	9.47	10.24	4.06	4.14	2.24	2.25	1.35	1.35
	50	26.32	35.16	16.83	19.90	9.60	10.39	4.04	4.12	2.26	2.26	1.35	1.35
	200	26.26	35.11	16.87	19.99	9.55	10.33	4.08	4.16	2.26	2.27	1.35	1.35
	500	25.37	34.18	16.41	19.35	9.55	10.34	4.05	4.13	2.24	2.25	1.35	1.35
0.95	10	29.52	39.78	18.81	22.26	10.59	11.47	4.54	4.63	2.52	2.53	1.50	1.50
	20	29.23	39.04	18.30	21.64	10.68	11.55	4.57	4.66	2.52	2.53	1.51	1.51
	30	28.55	38.23	18.72	22.07	10.46	11.32	4.56	4.65	2.52	2.53	1.51	1.51
	50	28.81	38.45	18.58	21.91	10.63	11.49	4.54	4.63	2.52	2.53	1.51	1.51
	200	27.91	36.84	18.67	22.04	10.67	11.53	4.54	4.63	2.52	2.53	1.51	1.51
	500	27.28	36.19	18.10	21.37	10.58	11.45	4.54	4.63	2.53	2.54	1.51	1.51

The algorithm that has been used to compute the MSEs and REs of the proposed estimators is given as:

1. Generating a population of size 5000 make use of bivariate normal distribution with $\left(Y,X\right) \sim {N}_{2}(2, 10, 1, 1,\rho )$.
2. Pick the value of ${\lambda }_{1}$ by fixing ${\lambda }_{2}$.
3. Total 50,000 samples of different sizes are considered i.e. 10, 20, 30, 50, 200, and 500.
4. Samples of step 3 are used, the 50,000 values are obtained for each estimator.
5. MSE is calculated for each sample size reported in Table 1.
6. The REs for each sample is computed by using (20) and given in Table 2.

## Discussion

The computed results for mean squared errors (MSEs) and relative errors (REs) are reported in Tables 1, 2, 3, 4, 5, 6, 7 and 8. These tables provide a comparison between the proposed memory type ratio estimators and the previous memory type estimator. Specifically, Tables 1, 2, 3 and 4 showcase the comparison between the proposed memory type ratio estimators and the previous memory type estimator. The values for the proposed product estimators are shown in Tables 5, 6, 7 and 8, respectively. The key findings for the proposed memory type estimators are provided below:

- It is observed that the MSEs are smaller and the REs are larger compared to the memory type ratio estimators in Tables 1, 2, 3 and 4. This demonstrates the efficiency of the proposed estimator over the previous one. Similar calculations from Tables 5, 6, 7 and 8 pertaining to the product estimators confirm the efficiency of the proposed product estimator over the previous memory type estimator.
- The correlation coefficient (ρ) between the study and auxiliary variable increases from 0 to 0.95, resulting in reduced MSE values and improved efficiency of the proposed memory type ratio estimator. In the case of the proposed product estimator, the value of ρ decreases from 0 to − 0.95, indicating an increase in estimator efficiency, as shown in Tables 5, 6, 7 and 8. Thus, the use of auxiliary information enhances the efficiency of the estimators.
- As the sample size increases while keeping λ_2_ and ρ fixed, different values of n are chosen, such as 10, 20, 30, 50, 200, and 500. The MSE values decrease with an increase in the sample size for each value of n. The values of the prediction relative errors (PRE) are consistently good for all n. Therefore, it can be concluded that the proposed memory type estimators are efficient for all values of n.
- The weights λ_1_ and λ_2_ are employed to assign weights to the current and previous sample values, thereby improving the efficiency of the proposed estimator, as evident in Tables 5, 6, 7 and 8. Ultimately, when λ_1_ = 1, no weight is given to past values, and the proposed estimators based on exponentially weighted moving average (EEWMA) depend solely on the current observation, similar to the previous ratio and product estimators.
- Hence, the proposed estimators perform equally well as the previous estimators based on exponentially weighted moving average (EWMA) for λ_1_ = 1, as shown in the last columns of Tables 5, 6, 7 and 8. Conversely, as the value of λ_1_ decreases, a larger weight is assigned to past sample values, leading to a gradual increase in the efficiency of the proposed estimator, as observed in Tables 5, 6, 7 and 8.

**Table 3:** MSEs of ratio estimators for ${\lambda }_{2}$=0.05.

$\lambda_{1}$		$0.1$		$0.15$		$0.25$		$0.5$		$0.75$		$1$	
**Ρ**	**n**	$$\overset{\lower0.5em\hbox{$\smash{\scriptscriptstyle\frown}$}}{t}_{rmi}$$	$$\overset{\lower0.5em\hbox{$\smash{\scriptscriptstyle\frown}$}}{t}_{ermi}$$	$$\overset{\lower0.5em\hbox{$\smash{\scriptscriptstyle\frown}$}}{t}_{rmi}$$	$$\overset{\lower0.5em\hbox{$\smash{\scriptscriptstyle\frown}$}}{t}_{ermi}$$	$$\overset{\lower0.5em\hbox{$\smash{\scriptscriptstyle\frown}$}}{t}_{rmi}$$	$$\overset{\lower0.5em\hbox{$\smash{\scriptscriptstyle\frown}$}}{t}_{ermi}$$	$$\overset{\lower0.5em\hbox{$\smash{\scriptscriptstyle\frown}$}}{t}_{rmi}$$	$$\overset{\lower0.5em\hbox{$\smash{\scriptscriptstyle\frown}$}}{t}_{ermi}$$	$$\overset{\lower0.5em\hbox{$\smash{\scriptscriptstyle\frown}$}}{t}_{rmi}$$	$$\overset{\lower0.5em\hbox{$\smash{\scriptscriptstyle\frown}$}}{t}_{ermi}$$	$$\overset{\lower0.5em\hbox{$\smash{\scriptscriptstyle\frown}$}}{t}_{rmi}$$	$$\overset{\lower0.5em\hbox{$\smash{\scriptscriptstyle\frown}$}}{t}_{ermi}$$
0.05	10	0.0054	0.0032	0.0084	0.0063	0.0145	0.0127	0.0337	0.0326	0.0606	0.0602	0.1005	0.1005
	20	0.0026	0.0015	0.0042	0.0031	0.0073	0.0064	0.0171	0.0165	0.0306	0.0304	0.0511	0.0511
	30	0.0017	0.0010	0.0028	0.0021	0.0048	0.0042	0.0115	0.0111	0.0202	0.0201	0.0339	0.0339
	50	0.0010	0.0006	0.0016	0.0012	0.0029	0.0025	0.0067	0.0065	0.0122	0.0122	0.0205	0.0205
	200	0.0002	0.0001	0.0004	0.0003	0.0007	0.0006	0.0016	0.0016	0.0030	0.0030	0.0050	0.0050
	500	0.0001	0.0000	0.0001	0.0001	0.0002	0.0002	0.0006	0.0006	0.0012	0.0012	0.0020	0.0020
0.25	10	0.0054	0.0028	0.0073	0.0054	0.0134	0.0117	0.0313	0.0303	0.0568	0.0564	0.0947	0.0947
	20	0.0024	0.0014	0.0038	0.0028	0.0066	0.0058	0.0157	0.0152	0.0282	0.0280	0.0471	0.0471
	30	0.0016	0.0009	0.0025	0.0018	0.0044	0.0038	0.0104	0.0101	0.0187	0.0186	0.0315	0.0315
	50	0.0009	0.0005	0.0015	0.0011	0.0027	0.0023	0.0062	0.0060	0.0112	0.0111	0.0186	0.0186
	200	0.0002	0.0001	0.0003	0.0002	0.0006	0.0005	0.0015	0.0014	0.0028	0.0028	0.0046	0.0046
	500	0.0000	0.0000	0.0001	0.0001	0.0002	0.0002	0.0006	0.0005	0.0011	0.0011	0.0018	0.0018
0.50	10	0.0040	0.0025	0.0067	0.0050	0.0119	0.0104	0.0276	0.0267	0.0507	0.0503	0.0839	0.0839
	20	0.0022	0.0013	0.0033	0.0024	0.0060	0.0053	0.0140	0.0135	0.0254	0.0252	0.0421	0.0421
	30	0.0014	0.0008	0.0022	0.0016	0.0040	0.0035	0.0092	0.0089	0.0167	0.0166	0.0280	0.0280
	50	0.0008	0.0005	0.0013	0.0010	0.0023	0.0020	0.0055	0.0054	0.0101	0.0101	0.0166	0.0166
	200	0.0002	0.0001	0.0003	0.0002	0.0006	0.0005	0.0014	0.0013	0.0025	0.0025	0.0041	0.0041
	500	0.0000	0.0000	0.0001	0.0001	0.0002	0.0002	0.0005	0.0005	0.0010	0.0009	0.0016	0.0016
0.75	10	0.0037	0.0021	0.0061	0.0046	0.0106	0.0092	0.0244	0.0236	0.0443	0.0440	0.0740	0.0740
	20	0.0019	0.0011	0.0029	0.0021	0.0052	0.0046	0.0123	0.0119	0.0224	0.0222	0.0370	0.0370
	30	0.0013	0.0007	0.0019	0.0014	0.0035	0.0030	0.0082	0.0080	0.0147	0.0146	0.0246	0.0246
	50	0.0007	0.0004	0.0012	0.0009	0.0021	0.0018	0.0049	0.0048	0.0088	0.0088	0.0148	0.0148
	200	0.0001	0.0001	0.0003	0.0002	0.0005	0.0004	0.0012	0.0012	0.0022	0.0021	0.0037	0.0037
	500	0.0000	0.0000	0.0001	0.0000	0.0002	0.0001	0.0004	0.0004	0.0008	0.0008	0.0014	0.0014
0.95	10	0.0033	0.0020	0.0053	0.0040	0.0097	0.0085	0.0222	0.0215	0.0400	0.0397	0.0662	0.0662
	20	0.0017	0.0010	0.0026	0.0020	0.0047	0.0041	0.0110	0.0106	0.0200	0.0199	0.0329	0.0329
	30	0.0011	0.0006	0.0017	0.0013	0.0032	0.0028	0.0072	0.0070	0.0132	0.0131	0.0221	0.0221
	50	0.0007	0.0004	0.0010	0.0007	0.0018	0.0016	0.0043	0.0042	0.0078	0.0078	0.0133	0.0133
	200	0.0001	0.0001	0.0002	0.0001	0.0004	0.0004	0.0010	0.0010	0.0019	0.0019	0.0033	0.0033
	500	0.0000	0.0000	0.0001	0.0000	0.0001	0.0001	0.0004	0.0004	0.0008	0.0007	0.0013	0.0013

**Table 4:** Relative efficiencies of ratio estimators for ${\lambda }_{2}$=0.05.

$\lambda_{1}$		$0.1$		$0.15$		$0.25$		$0.5$		$0.75$		$1$	
**Ρ**	**n**	$$\overset{\lower0.5em\hbox{$\smash{\scriptscriptstyle\frown}$}}{t}_{rmi}$$	$$\overset{\lower0.5em\hbox{$\smash{\scriptscriptstyle\frown}$}}{t}_{ermi}$$	$$\overset{\lower0.5em\hbox{$\smash{\scriptscriptstyle\frown}$}}{t}_{rmi}$$	$$\overset{\lower0.5em\hbox{$\smash{\scriptscriptstyle\frown}$}}{t}_{ermi}$$	$$\overset{\lower0.5em\hbox{$\smash{\scriptscriptstyle\frown}$}}{t}_{rmi}$$	$$\overset{\lower0.5em\hbox{$\smash{\scriptscriptstyle\frown}$}}{t}_{ermi}$$	$$\overset{\lower0.5em\hbox{$\smash{\scriptscriptstyle\frown}$}}{t}_{rmi}$$	$$\overset{\lower0.5em\hbox{$\smash{\scriptscriptstyle\frown}$}}{t}_{ermi}$$	$$\overset{\lower0.5em\hbox{$\smash{\scriptscriptstyle\frown}$}}{t}_{rmi}$$	$$\overset{\lower0.5em\hbox{$\smash{\scriptscriptstyle\frown}$}}{t}_{ermi}$$	$$\overset{\lower0.5em\hbox{$\smash{\scriptscriptstyle\frown}$}}{t}_{rmi}$$	$$\overset{\lower0.5em\hbox{$\smash{\scriptscriptstyle\frown}$}}{t}_{ermi}$$
0.05	10	18.38	30.99	11.90	15.86	6.86	7.84	2.96	3.06	1.63	1.64	0.98	0.98
	20	18.94	32.71	11.83	15.82	6.91	7.89	2.92	3.02	1.63	1.64	0.98	0.98
	30	18.88	31.98	11.83	15.79	6.79	7.77	2.93	3.03	1.64	1.65	0.98	0.98
	50	18.73	32.20	12.20	16.41	6.90	7.87	2.96	3.06	1.63	1.64	0.98	0.98
	200	18.99	32.95	12.10	16.16	6.93	7.92	2.95	3.05	1.62	1.63	0.98	0.98
	500	18.69	32.12	12.33	16.57	6.95	7.94	2.95	3.05	1.63	1.64	0.98	0.98
0.25	10	20.24	34.71	13.60	18.30	7.40	8.46	3.18	3.29	1.78	1.79	1.06	1.06
	20	20.45	34.89	12.96	17.37	7.51	8.59	3.20	3.30	1.77	1.78	1.06	1.06
	30	20.74	35.87	13.14	17.59	7.48	8.54	3.20	3.31	1.77	1.79	1.07	1.07
	50	20.22	34.72	13.08	17.59	7.40	8.44	3.20	3.31	1.78	1.79	1.07	1.07
	200	20.95	36.04	13.36	17.96	7.50	8.57	3.21	3.32	1.77	1.78	1.07	1.07
	500	20.96	36.20	13.01	17.31	7.43	8.50	3.21	3.32	1.77	1.78	1.07	1.07
0.50	10	22.54	38.97	14.81	19.77	8.36	9.58	3.56	3.68	1.98	1.99	1.18	1.18
	20	22.41	38.26	14.98	20.16	8.28	9.46	3.56	3.68	1.98	2.00	1.19	1.19
	30	22.61	38.92	14.80	19.88	8.21	9.38	3.57	3.68	1.99	2.00	1.19	1.19
	50	22.70	38.53	14.55	19.51	8.33	9.51	3.59	3.71	1.99	2.00	1.19	1.19
	200	22.28	37.69	14.40	19.18	8.27	9.47	3.57	3.69	1.97	1.99	1.19	1.19
	500	22.45	38.31	14.65	19.61	8.45	9.66	3.57	3.69	1.99	2.00	1.19	1.19
0.75	10	26.72	45.85	16.43	21.91	9.44	10.81	4.07	4.21	2.26	2.28	1.34	1.34
	20	25.40	43.70	17.06	22.82	9.45	10.80	4.05	4.18	2.24	2.26	1.35	1.35
	30	25.65	43.36	16.77	22.45	9.49	10.87	4.02	4.16	2.25	2.26	1.35	1.35
	50	25.94	44.77	16.40	21.96	9.38	10.71	4.03	4.16	2.25	2.26	1.35	1.35
	200	25.54	43.71	16.65	22.27	9.36	10.68	4.02	4.16	2.25	2.27	1.35	1.35
	500	26.06	45.03	16.62	22.31	9.43	10.80	4.04	4.18	2.25	2.27	1.35	1.35
0.95	10	29.55	50.11	18.71	25.04	10.36	11.82	4.51	4.66	2.51	2.52	1.50	1.50
	20	28.88	49.46	18.61	24.96	10.58	12.06	4.55	4.70	2.51	2.52	1.51	1.51
	30	29.20	50.41	18.64	25.07	10.46	11.93	4.56	4.71	2.51	2.53	1.51	1.51
	50	28.62	48.69	18.83	25.26	10.56	12.08	4.57	4.71	2.53	2.55	1.51	1.51
	200	28.88	49.51	18.85	25.11	10.42	11.90	4.56	4.71	2.53	2.54	1.51	1.51
	500	29.44	50.85	18.85	25.27	10.66	12.18	4.61	4.76	2.53	2.55	1.51	1.51

**Table 5:** MSEs of product estimators for ${\lambda }_{2}$=0.03.

$\lambda_{1}$		$0.1$		$0.15$		$0.25$		$0.5$		$0.75$		$1$	
**Ρ**	**n**	$$\overset{\lower0.5em\hbox{$\smash{\scriptscriptstyle\frown}$}}{t}_{rmi}$$	$$\overset{\lower0.5em\hbox{$\smash{\scriptscriptstyle\frown}$}}{t}_{ermi}$$	$$\overset{\lower0.5em\hbox{$\smash{\scriptscriptstyle\frown}$}}{t}_{rmi}$$	$$\overset{\lower0.5em\hbox{$\smash{\scriptscriptstyle\frown}$}}{t}_{ermi}$$	$$\overset{\lower0.5em\hbox{$\smash{\scriptscriptstyle\frown}$}}{t}_{rmi}$$	$$\overset{\lower0.5em\hbox{$\smash{\scriptscriptstyle\frown}$}}{t}_{ermi}$$	$$\overset{\lower0.5em\hbox{$\smash{\scriptscriptstyle\frown}$}}{t}_{rmi}$$	$$\overset{\lower0.5em\hbox{$\smash{\scriptscriptstyle\frown}$}}{t}_{ermi}$$	$$\overset{\lower0.5em\hbox{$\smash{\scriptscriptstyle\frown}$}}{t}_{rmi}$$	$$\overset{\lower0.5em\hbox{$\smash{\scriptscriptstyle\frown}$}}{t}_{ermi}$$	$$\overset{\lower0.5em\hbox{$\smash{\scriptscriptstyle\frown}$}}{t}_{rmi}$$	$$\overset{\lower0.5em\hbox{$\smash{\scriptscriptstyle\frown}$}}{t}_{ermi}$$
− 0.05	10	0.0038	0.0028	0.0082	0.0069	0.0148	0.0137	0.0339	0.0332	0.0624	0.0621	0.1030	0.1030
	20	0.0019	0.0014	0.0041	0.0035	0.0072	0.0066	0.0169	0.0166	0.0305	0.0304	0.0511	0.0511
	30	0.0012	0.0009	0.0027	0.0023	0.0047	0.0044	0.0111	0.0109	0.0204	0.0203	0.0338	0.0338
	50	0.0007	0.0005	0.0016	0.0013	0.0028	0.0026	0.0068	0.0066	0.0123	0.0122	0.0204	0.0204
	200	0.0002	0.0001	0.0004	0.0003	0.0007	0.0006	0.0017	0.0016	0.0030	0.0030	0.0051	0.0051
	500	0.0000	0.0000	0.0001	0.0001	0.0003	0.0002	0.0006	0.0006	0.0012	0.0012	0.0020	0.0020
− 0.25	10	0.0049	0.0036	0.0078	0.0066	0.0131	0.0121	0.0309	0.0303	0.0572	0.0570	0.0946	0.0946
	20	0.0024	0.0018	0.0037	0.0031	0.0068	0.0062	0.0156	0.0153	0.0282	0.0281	0.0463	0.0463
	30	0.0016	0.0012	0.0024	0.0021	0.0044	0.0041	0.0104	0.0101	0.0186	0.0186	0.0314	0.0314
	50	0.0009	0.0007	0.0015	0.0012	0.0027	0.0025	0.0062	0.0061	0.0112	0.0112	0.0187	0.0187
	200	0.0002	0.0001	0.0004	0.0003	0.0006	0.0006	0.0015	0.0015	0.0028	0.0027	0.0047	0.0047
	500	0.0000	0.0000	0.0001	0.0001	0.0002	0.0002	0.0006	0.0006	0.0011	0.0011	0.0018	0.0018
− 0.50	10	0.0043	0.0032	0.0067	0.0057	0.0120	0.0111	0.0283	0.0277	0.0502	0.0500	0.0842	0.0842
	20	0.0026	0.0017	0.0034	0.0028	0.0059	0.0054	0.0138	0.0135	0.0251	0.0250	0.0421	0.0421
	30	0.0014	0.0011	0.0022	0.0018	0.0040	0.0037	0.0093	0.0091	0.0167	0.0167	0.0278	0.0278
	50	0.0009	0.0006	0.0013	0.0011	0.0023	0.0021	0.0056	0.0054	0.0101	0.0100	0.0167	0.0167
	200	0.0002	0.0001	0.0003	0.0002	0.0006	0.0005	0.0013	0.0013	0.0025	0.0025	0.0041	0.0041
	500	0.0000	0.0000	0.0001	0.0001	0.0002	0.0002	0.0005	0.0005	0.0010	0.0009	0.0016	0.0016
− 0.75	10	0.0038	0.0028	0.0060	0.0051	0.0107	0.0098	0.0245	0.0240	0.0445	0.0443	0.0739	0.0739
	20	0.0019	0.0014	0.0029	0.0025	0.0052	0.0048	0.0124	0.0121	0.0224	0.0223	0.0370	0.0370
	30	0.0012	0.0009	0.0020	0.0017	0.0035	0.0032	0.0081	0.0079	0.0148	0.0147	0.0248	0.0248
	50	0.0007	0.0005	0.0011	0.0009	0.0020	0.0019	0.0049	0.0048	0.0088	0.0088	0.0147	0.0147
	200	0.0002	0.0001	0.0003	0.0002	0.0005	0.0004	0.0012	0.0012	0.0022	0.0022	0.0036	0.0036
	500	0.0000	0.0000	0.0001	0.0001	0.0002	0.0001	0.0004	0.0004	0.0008	0.0008	0.0014	0.0014
− 0.95	10	0.0035	0.0026	0.0053	0.0045	0.0093	0.0086	0.0219	0.0214	0.0397	0.0395	0.0658	0.0658
	20	0.0017	0.0013	0.0026	0.0022	0.0047	0.0043	0.0110	0.0108	0.0196	0.0195	0.0329	0.0329
	30	0.0011	0.0008	0.0018	0.0015	0.0031	0.0029	0.0074	0.0072	0.0132	0.0132	0.0218	0.0218
	50	0.0007	0.0005	0.0010	0.0009	0.0018	0.0017	0.0044	0.0043	0.0078	0.0078	0.0132	0.0132
	200	0.0002	0.0001	0.0002	0.0002	0.0004	0.0004	0.0011	0.0010	0.0019	0.0019	0.0033	0.0033
	500	0.0000	0.0000	0.0001	0.0000	0.0001	0.0001	0.0004	0.0004	0.0007	0.0007	0.0013	0.0013

**Table 6:** Relative efficiencies of product estimators for ${\lambda }_{2}$=0.03.

$\lambda_{1}$		$0.1$		$0.15$		$0.25$		$0.5$		$0.75$		$1$	
**Ρ**	**n**	$$\overset{\lower0.5em\hbox{$\smash{\scriptscriptstyle\frown}$}}{t}_{rmi}$$	$$\overset{\lower0.5em\hbox{$\smash{\scriptscriptstyle\frown}$}}{t}_{ermi}$$	$$\overset{\lower0.5em\hbox{$\smash{\scriptscriptstyle\frown}$}}{t}_{rmi}$$	$$\overset{\lower0.5em\hbox{$\smash{\scriptscriptstyle\frown}$}}{t}_{ermi}$$	$$\overset{\lower0.5em\hbox{$\smash{\scriptscriptstyle\frown}$}}{t}_{rmi}$$	$$\overset{\lower0.5em\hbox{$\smash{\scriptscriptstyle\frown}$}}{t}_{ermi}$$	$$\overset{\lower0.5em\hbox{$\smash{\scriptscriptstyle\frown}$}}{t}_{rmi}$$	$$\overset{\lower0.5em\hbox{$\smash{\scriptscriptstyle\frown}$}}{t}_{ermi}$$	$$\overset{\lower0.5em\hbox{$\smash{\scriptscriptstyle\frown}$}}{t}_{rmi}$$	$$\overset{\lower0.5em\hbox{$\smash{\scriptscriptstyle\frown}$}}{t}_{ermi}$$	$$\overset{\lower0.5em\hbox{$\smash{\scriptscriptstyle\frown}$}}{t}_{rmi}$$	$$\overset{\lower0.5em\hbox{$\smash{\scriptscriptstyle\frown}$}}{t}_{ermi}$$
− 0.05	10	25.85	34.67	12.15	14.33	6.73	7.27	2.94	3.00	1.63	1.64	0.98	0.98
	20	25.99	34.96	11.92	14.00	6.91	7.48	2.94	3.00	1.64	1.64	0.98	0.98
	30	26.47	35.50	12.17	14.34	6.87	7.42	2.94	3.00	1.64	1.65	0.98	0.98
	50	26.02	34.94	12.17	14.40	6.95	7.53	2.91	2.97	1.62	1.63	0.98	0.98
	200	25.32	33.65	12.15	14.34	6.92	7.50	2.94	3.00	1.64	1.65	0.98	0.98
	500	24.84	32.91	12.09	14.26	6.72	7.27	2.94	3.00	1.64	1.65	0.98	0.98
− 0.25	10	20.29	27.20	12.81	15.13	7.58	8.20	3.21	3.27	1.77	1.78	1.06	1.06
	20	20.77	27.81	13.34	15.75	7.35	7.95	3.18	3.24	1.76	1.77	1.06	1.06
	30	20.59	27.48	13.42	15.87	7.56	8.18	3.21	3.27	1.78	1.78	1.06	1.06
	50	20.60	27.54	13.27	15.64	7.28	7.87	3.21	3.28	1.77	1.78	1.06	1.06
	200	20.45	27.35	12.78	15.08	7.34	7.94	3.21	3.27	1.78	1.79	1.06	1.06
	500	20.19	26.99	13.23	15.63	7.38	7.99	3.17	3.23	1.78	1.79	1.06	1.06
− 0.50	10	22.96	30.87	14.92	17.65	8.27	8.96	3.54	3.61	1.98	1.99	1.19	1.19
	20	22.22	29.56	14.68	17.34	8.40	9.09	3.60	3.67	1.98	1.99	1.19	1.19
	30	22.69	30.12	14.82	17.52	8.20	8.86	3.59	3.67	1.99	2.00	1.19	1.19
	50	21.73	29.04	14.41	17.04	8.39	9.08	3.56	3.63	1.98	1.99	1.19	1.19
	200	22.24	29.50	14.29	16.83	8.36	9.04	3.59	3.66	1.99	2.00	1.19	1.19
	500	22.81	30.53	14.57	17.24	8.38	9.06	3.58	3.65	1.98	1.99	1.19	1.19
− 0.75	10	25.97	34.55	16.56	19.65	9.33	10.09	4.06	4.14	2.24	2.25	1.35	1.35
	20	25.63	34.41	16.82	19.83	9.50	10.28	4.04	4.12	2.25	2.26	1.35	1.35
	30	26.02	34.80	16.48	19.48	9.41	10.18	4.07	4.15	2.25	2.26	1.35	1.35
	50	25.08	33.39	17.20	20.37	9.54	10.33	4.03	4.11	2.26	2.27	1.35	1.35
	200	25.81	34.37	16.60	19.64	9.45	10.24	4.03	4.11	2.25	2.26	1.35	1.35
	500	24.88	33.06	16.50	19.46	9.35	10.12	4.05	4.14	2.27	2.27	1.35	1.35
− 0.95	10	28.16	37.71	18.54	21.86	10.70	11.59	4.55	4.64	2.53	2.54	1.51	1.51
	20	28.60	38.08	18.62	21.92	10.54	11.39	4.49	4.58	2.52	2.53	1.51	1.51
	30	29.86	40.17	18.27	21.54	10.52	11.38	4.52	4.62	2.52	2.53	1.51	1.51
	50	28.21	37.61	18.28	21.61	10.59	11.46	4.53	4.62	2.53	2.53	1.51	1.51
	200	28.86	38.56	18.59	21.98	10.49	11.35	4.52	4.61	2.52	2.53	1.51	1.51
	500	28.97	38.52	18.61	21.97	10.80	11.69	4.53	4.62	2.52	2.53	1.51	1.51

**Table 7:** MSEs of product estimator for ${\lambda }_{2}$=0.05.

$\lambda_{1}$		$0.1$		$0.15$		$0.25$		$0.5$		$0.75$		$1$	
**Ρ**	**n**	$$\overset{\lower0.5em\hbox{$\smash{\scriptscriptstyle\frown}$}}{t}_{rmi}$$	$$\overset{\lower0.5em\hbox{$\smash{\scriptscriptstyle\frown}$}}{t}_{ermi}$$	$$\overset{\lower0.5em\hbox{$\smash{\scriptscriptstyle\frown}$}}{t}_{rmi}$$	$$\overset{\lower0.5em\hbox{$\smash{\scriptscriptstyle\frown}$}}{t}_{ermi}$$	$$\overset{\lower0.5em\hbox{$\smash{\scriptscriptstyle\frown}$}}{t}_{rmi}$$	$$\overset{\lower0.5em\hbox{$\smash{\scriptscriptstyle\frown}$}}{t}_{ermi}$$	$$\overset{\lower0.5em\hbox{$\smash{\scriptscriptstyle\frown}$}}{t}_{rmi}$$	$$\overset{\lower0.5em\hbox{$\smash{\scriptscriptstyle\frown}$}}{t}_{ermi}$$	$$\overset{\lower0.5em\hbox{$\smash{\scriptscriptstyle\frown}$}}{t}_{rmi}$$	$$\overset{\lower0.5em\hbox{$\smash{\scriptscriptstyle\frown}$}}{t}_{ermi}$$	$$\overset{\lower0.5em\hbox{$\smash{\scriptscriptstyle\frown}$}}{t}_{rmi}$$	$$\overset{\lower0.5em\hbox{$\smash{\scriptscriptstyle\frown}$}}{t}_{ermi}$$
− 0.05	10	0.0054	0.0031	0.0080	0.0060	0.0141	0.0123	0.0336	0.0325	0.0612	0.0608	0.1012	0.1012
	20	0.0027	0.0016	0.0040	0.0030	0.0072	0.0063	0.0170	0.0165	0.0305	0.0303	0.0509	0.0509
	30	0.0017	0.0010	0.0028	0.0021	0.0048	0.0042	0.0113	0.0110	0.0203	0.0202	0.0337	0.0337
	50	0.0010	0.0006	0.0016	0.0012	0.0029	0.0025	0.0068	0.0066	0.0122	0.0121	0.0203	0.0203
	200	0.0002	0.0001	0.0004	0.0003	0.0007	0.0006	0.0017	0.0016	0.0030	0.0030	0.0051	0.0051
	500	0.0001	0.0000	0.0001	0.0001	0.0003	0.0002	0.0006	0.0006	0.0012	0.0012	0.0020	0.0020
− 0.25	10	0.0048	0.0028	0.0074	0.0055	0.0133	0.0116	0.0310	0.0300	0.0566	0.0563	0.0933	0.0933
	20	0.0024	0.0014	0.0038	0.0028	0.0067	0.0059	0.0154	0.0149	0.0284	0.0282	0.0472	0.0472
	30	0.0016	0.0009	0.0026	0.0019	0.0044	0.0038	0.0103	0.0100	0.0189	0.0188	0.0313	0.0313
	50	0.0009	0.0005	0.0015	0.0011	0.0026	0.0023	0.0062	0.0060	0.0113	0.0112	0.0190	0.0190
	200	0.0002	0.0001	0.0003	0.0002	0.0006	0.0006	0.0015	0.0015	0.0028	0.0027	0.0046	0.0046
	500	0.0000	0.0000	0.0001	0.0001	0.0002	0.0002	0.0006	0.0006	0.0011	0.0011	0.0018	0.0018
− 0.50	10	0.0050	0.0026	0.0068	0.0051	0.0119	0.0104	0.0279	0.0270	0.0503	0.0500	0.0840	0.0840
	20	0.0022	0.0013	0.0034	0.0025	0.0059	0.0051	0.0140	0.0136	0.0254	0.0253	0.0420	0.0420
	30	0.0014	0.0008	0.0022	0.0016	0.0039	0.0034	0.0093	0.0090	0.0167	0.0166	0.0279	0.0279
	50	0.0009	0.0005	0.0013	0.0009	0.0023	0.0020	0.0055	0.0053	0.0101	0.0100	0.0167	0.0167
	200	0.0002	0.0001	0.0003	0.0002	0.0006	0.0005	0.0013	0.0013	0.0025	0.0025	0.0042	0.0042
	500	0.0000	0.0000	0.0001	0.0001	0.0002	0.0002	0.0005	0.0005	0.0010	0.0009	0.0016	0.0016
− 0.75	10	0.0040	0.0023	0.0060	0.0045	0.0108	0.0095	0.0244	0.0236	0.0443	0.0440	0.0742	0.0742
	20	0.0019	0.0011	0.0029	0.0021	0.0053	0.0046	0.0120	0.0116	0.0222	0.0221	0.0370	0.0370
	30	0.0013	0.0007	0.0019	0.0014	0.0034	0.0030	0.0081	0.0078	0.0148	0.0147	0.0246	0.0246
	50	0.0008	0.0004	0.0011	0.0008	0.0020	0.0018	0.0049	0.0047	0.0089	0.0088	0.0147	0.0147
	200	0.0001	0.0001	0.0002	0.0002	0.0005	0.0004	0.0012	0.0011	0.0022	0.0022	0.0036	0.0036
	500	0.0000	0.0000	0.0001	0.0000	0.0002	0.0001	0.0004	0.0004	0.0008	0.0008	0.0014	0.0014
− 0.95	10	0.0034	0.0020	0.0052	0.0039	0.0096	0.0084	0.0220	0.0213	0.0396	0.0393	0.0660	0.0660
	20	0.0017	0.0010	0.0027	0.0020	0.0047	0.0041	0.0110	0.0106	0.0197	0.0196	0.0330	0.0330
	30	0.0011	0.0006	0.0017	0.0013	0.0031	0.0027	0.0073	0.0071	0.0132	0.0131	0.0218	0.0218
	50	0.0006	0.0003	0.0010	0.0008	0.0018	0.0016	0.0043	0.0042	0.0079	0.0078	0.0130	0.0130
	200	0.0001	0.0001	0.0002	0.0002	0.0004	0.0004	0.0011	0.0010	0.0019	0.0019	0.0033	0.0033
	500	0.0000	0.0000	0.0001	0.0000	0.0001	0.0001	0.0004	0.0004	0.0007	0.0007	0.0013	0.0013

**Table 8:** Relative efficiencies of product estimators for ${\lambda }_{2}$=0.05.

$\lambda_{1}$		$0.1$		$0.15$		$0.25$		$0.5$		$0.75$		$1$	
**Ρ**	**n**	$$\overset{\lower0.5em\hbox{$\smash{\scriptscriptstyle\frown}$}}{t}_{rmi}$$	$$\overset{\lower0.5em\hbox{$\smash{\scriptscriptstyle\frown}$}}{t}_{ermi}$$	$$\overset{\lower0.5em\hbox{$\smash{\scriptscriptstyle\frown}$}}{t}_{rmi}$$	$$\overset{\lower0.5em\hbox{$\smash{\scriptscriptstyle\frown}$}}{t}_{ermi}$$	$$\overset{\lower0.5em\hbox{$\smash{\scriptscriptstyle\frown}$}}{t}_{rmi}$$	$$\overset{\lower0.5em\hbox{$\smash{\scriptscriptstyle\frown}$}}{t}_{ermi}$$	$$\overset{\lower0.5em\hbox{$\smash{\scriptscriptstyle\frown}$}}{t}_{rmi}$$	$$\overset{\lower0.5em\hbox{$\smash{\scriptscriptstyle\frown}$}}{t}_{ermi}$$	$$\overset{\lower0.5em\hbox{$\smash{\scriptscriptstyle\frown}$}}{t}_{rmi}$$	$$\overset{\lower0.5em\hbox{$\smash{\scriptscriptstyle\frown}$}}{t}_{ermi}$$	$$\overset{\lower0.5em\hbox{$\smash{\scriptscriptstyle\frown}$}}{t}_{rmi}$$	$$\overset{\lower0.5em\hbox{$\smash{\scriptscriptstyle\frown}$}}{t}_{ermi}$$
− 0.05	10	18.43	31.67	12.38	16.53	7.05	8.08	2.95	3.05	1.63	1.64	0.98	0.98
	20	18.45	31.16	12.27	16.44	6.83	7.80	2.94	3.04	1.63	1.64	0.98	0.98
	30	18.56	31.87	11.88	15.91	6.89	7.89	2.95	3.04	1.64	1.65	0.98	0.98
	50	18.78	32.34	12.01	16.04	6.84	7.81	2.94	3.04	1.63	1.64	0.98	0.98
	200	18.45	31.37	11.74	15.66	6.88	7.86	2.93	3.03	1.63	1.64	0.98	0.98
	500	18.40	31.31	12.11	16.17	6.85	7.84	2.92	3.02	1.64	1.65	0.98	0.98
− 0.25	10	20.69	35.38	13.36	17.87	7.47	8.53	3.21	3.32	1.78	1.79	1.06	1.06
	20	20.41	35.40	13.05	17.49	7.34	8.40	3.25	3.35	1.77	1.79	1.06	1.06
	30	20.74	35.82	12.73	17.04	7.55	8.62	3.21	3.31	1.77	1.79	1.06	1.06
	50	20.28	34.81	12.99	17.37	7.45	8.52	3.19	3.29	1.77	1.78	1.06	1.06
	200	20.95	36.15	13.34	17.95	7.27	8.30	3.19	3.30	1.77	1.78	1.06	1.06
	500	20.07	34.29	13.41	18.06	7.47	8.54	3.19	3.30	1.77	1.78	1.06	1.06
− 0.50	10	22.11	37.08	14.74	19.62	8.30	9.49	3.59	3.71	1.99	2.00	1.19	1.19
	20	22.19	37.94	14.57	19.53	8.41	9.62	3.56	3.68	1.98	1.99	1.19	1.19
	30	23.34	39.86	14.73	19.81	8.41	9.61	3.57	3.69	1.99	2.00	1.19	1.19
	50	22.02	37.23	15.06	20.26	8.35	9.58	3.59	3.70	1.98	1.99	1.20	1.20
	200	22.54	38.79	14.51	19.41	8.23	9.42	3.55	3.67	1.97	1.99	1.19	1.19
	500	22.44	38.26	14.44	19.26	8.21	9.38	3.56	3.68	1.99	2.00	1.19	1.19
− 0.75	10	25.21	43.08	16.48	21.99	9.24	10.55	4.06	4.20	2.25	2.26	1.35	1.35
	20	25.27	43.43	17.00	22.77	9.39	10.73	4.08	4.22	2.24	2.25	1.35	1.35
	30	25.86	44.94	17.11	22.94	9.52	10.89	4.08	4.22	2.26	2.27	1.35	1.35
	50	25.00	42.43	16.69	22.38	9.48	10.83	4.02	4.16	2.25	2.26	1.35	1.35
	200	25.09	42.28	16.78	22.42	9.45	10.79	4.05	4.19	2.26	2.27	1.35	1.35
	500	26.14	44.27	16.72	22.34	9.50	10.86	4.07	4.20	2.25	2.27	1.35	1.35
− 0.95	10	28.71	48.93	18.91	25.31	10.51	12.01	4.58	4.73	2.54	2.55	1.51	1.51
	20	28.32	48.47	18.51	24.71	10.60	12.10	4.58	4.73	2.52	2.54	1.51	1.51
	30	29.22	50.48	18.76	25.18	10.55	12.03	4.52	4.68	2.52	2.54	1.51	1.51
	50	29.60	50.84	18.50	24.77	10.63	12.17	4.55	4.70	2.52	2.54	1.52	1.52
	200	28.37	48.28	18.64	24.96	10.61	12.13	4.49	4.64	2.53	2.55	1.52	1.52
	500	29.04	50.09	18.28	24.24	10.68	12.21	4.59	4.74	2.53	2.54	1.51	1.51

## Mathematical comparison

The memory type proposed estimators are recommended for use in real life as compared to the previous estimators if they have lesser mean square errors. In this section, we explained the conditions under which the proposed memory estimator performs well than previous memory type estimators. the proposed memory type will be more efficient than the previous one if:

$$MSE\left({\widehat{t}}_{ermi}\right)< MSE\left({\widehat{t}}_{rmi}\right),$$

$$\theta \gamma \left[{C}_{y}^{2}+{C}_{x}^{2}-2\rho {C}_{y}{C}_{x}\right]< \theta \frac{\lambda }{2-\lambda }\left[{C}_{y}^{2}+{C}_{x}^{2}-2\rho {C}_{y}{C}_{x}\right],$$

$$\gamma < \frac{\lambda }{2-\lambda },$$

21 $$\frac{\gamma (2-\lambda )}{\lambda }<1,$$

The condition for the proposed product estimator is:

$$MSE\left({\widehat{\overline{Y}} }_{epmi}\right)< MSE\left({\widehat{\overline{Y}} }_{pmi}\right),$$

$$\theta \gamma \left[{C}_{y}^{2}+{C}_{x}^{2}+2\rho {C}_{y}{C}_{x}\right]< \theta \frac{\lambda }{2-\lambda }\left[{C}_{y}^{2}+{C}_{x}^{2}+2\rho {C}_{y}{C}_{x}\right],$$

$$\gamma < \frac{\lambda }{2-\lambda },$$

22 $$\frac{\gamma (2-\lambda )}{\lambda }<1,$$

which will always be the case unless ${\lambda }_{1}< {\lambda }_{2}$. Hence, the proposed memory type ratio and product estimators are preferable and more efficient than the previous ones.

## Real data application

In this section, we apply the proposed estimator to a real data set to demonstrate its practical application. The data set used in this illustration is obtained from the agricultural statistics reports of Pakistan, specifically from the department of research and national food security. The data set focuses on the yield of wheat, denoted as variable Y, measured in kilograms, and the corresponding area of cultivation, denoted as variable X. By analyzing this real data set, we can assess the performance and effectiveness of the proposed ratio estimator in estimating the relationship between wheat yield and cultivation area. This application allows us to evaluate the practical utility of the proposed estimator in the context of agricultural statistics in Pakistan.

The values of the population average for variables y and x are attained as ${\mu }_{y }=$ 2545.4 and ${\mu }_{x }=$ 6341.2 by taking the average of all sample mean values. The mean per unit estimator is attained from the agricultural report as $\overline{y }$ and $\overline{x }$. The EEWMA statistic is evaluated from each sample with ${\lambda }_{1}$ = 0.25 and ${\lambda }_{2}$ = 0.05. The Table 9 presents the computation of the proposed ratio type estimator and the estimated values of variables Y and X. From the Table 9 we observed that proposed EEWMA ratio estimator provides more smoothed estimation as compared to the comparative one. This shows that using of EEWMA provides more efficient estimate with respect to the time.

**Table 9:** Computation of proposed memory type ratio estimator.

Year	$\overline{x }$	$\overline{y }$	${\widehat{t}}_{rmi}$	${Z}_{i}$	${Q}_{i}$	${\widehat{t`}}_{ermi}$
1996	5973.5	2081	2513.49	2429.30	6249.27	2465.04
1997	5839.9	2118	2493.59	2368.89	6160.72	2438.29
1998	5934.6	2327	2492.86	2370.96	6120.23	2456.57
1999	5934.6	2226	2481.93	2336.92	6083.10	2436.07
2000	6180.3	2667	2507.27	2424.99	6114.83	2514.76
2001	6255.5	2465	2506.40	2422.89	6146.72	2499.55
2002	6101.8	2392	2504.36	2413.06	6130.05	2496.18
2003	6097.3	2518	2515.67	2440.35	6123.28	2527.20
2004	6255.5	2500	2517.56	2451.38	6157.63	2524.46
2005	6378.9	2724	2537.09	2517.10	6208.06	2571.09
2006	6483.4	2588	2536.44	2524.48	6268.35	2553.82
2007	6432.8	2775	2556.94	2583.94	6298.71	2601.37
2008	6402.0	2438	2542.41	2537.90	6317.83	2547.29
2009	6836.2	2694	2537.76	2581.92	6443.21	2541.04
2010	6913.5	2592	2520.40	2578.84	6541.13	2500.01
2011	6691.0	2846	2538.81	2644.97	6559.98	2556.76
2012	6482.9	2736	2552.71	2657.67	6534.16	2579.19
2013	6511.3	2855	2575.72	2703.09	6531.01	2624.53
2014	6778.4	2821	2582.37	2724.97	6593.84	2620.56

## Conclusion

Sampling techniques give several estimation methods to enrich the efficiency of the estimators. The developed estimators use only current sample information. In this paper, we have proposed the ratio and product type estimators in the form of EEWMA statistic which incorporates the previous sample data with the current information. Based on the results of PREs given in Tables 5, 6, 7 and 8, it may be concluded that the proposed memory type estimators are more efficient to estimate the population mean as compared to the previous memory type estimator based on EWMA statistic. In the current study, we utilized a single auxiliary variable for the estimation. More than one auxiliary variable can be used for the estimation. Furthermore, the study can be extended to other sampling designs.

## Data Availability

The datasets used and/or analyzed during the current study are available from the corresponding author upon reasonable request. Further, no experiments on humans and/or the use of human tissue samples involved in this study.
